# Case Report: Acute Thrombotic Angiopathy of Atrial Appendage Epicardial Veins: A Seemingly Innocuous Finding Portending a Fatal Outcome

**DOI:** 10.3389/fcvm.2021.621632

**Published:** 2021-03-10

**Authors:** Simona Pichler Sekulic, Miroslav Sekulic

**Affiliations:** Department of Pathology and Cell Biology, Columbia University Irving Medical Center, New York, NY, United States

**Keywords:** atrial appendage, acute thrombotic angiopathy, thrombosis, pulmonary embolism, atrial fibrillation

## Abstract

Thrombotic angiopathy is a pathologic description to describe endothelial injury, and with sufficient and sustained injury can lead to exposure of underlying tissue factor and the deposition of associated fibrin material. We present briefly a case of an 87-year-old woman with mitral valve regurgitation and atrial fibrillation undergoing mitral valve annuloplasty, Cox-maze procedure, and excision of the left atrial appendage. Pathologic examination of the excised atrial appendage revealed commonly encountered cardiomyocyte hypertrophy and endocardial fibroelastosis, however also showed a non-occlusive, acute thrombotic angiopathy involving epicardial veins. The surgical and immediate post-operative course was unremarkable; however, 3 weeks after discharge, the patient would develop a fatal pulmonary embolism. While fibrin thrombosis developing within the atrial appendage chamber is a recognized concern in the setting of atrial fibrillation, the significance of an acute thrombotic angiopathy involving epicardial veins of the atrial appendage is less clear although in the presented case was the sole potential harbinger of a subsequent fatal thrombotic event.

## Introduction

In patients with atrial fibrillation, the risk of formation of fibrin thrombi within the chamber that could lead to thromboembolic events is in part the impetus for surgical intervention (1, 2). While the care of patients with atrial fibrillation includes non-surgical medical management with rate control and antithrombotic/anticoagulant therapy, surgical intervention is required in some to reduce the risk of such thromboembolic events (3). Examination of atrial appendages removed in the setting of surgical management of atrial fibrillation may occasionally reveal an associated endocardial thrombus within the chamber and described histopathological changes including cardiomyocyte hypertrophy, interstitial fibrosis, and endocardial fibroelastosis (1).

Herein, we present a case of a patient undergoing excision of the left atrial appendage in the setting of mitral valve regurgitation and atrial fibrillation, with the excised tissue revealing few epicardial veins with a non-occlusive acute thrombotic angiopathy. While unique, the managing clinical team was not concerned by the finding, and the patient was discharged after an unremarkable post-operative course, however presented 3 weeks later with a fatal pulmonary embolism. The initially innocuous finding of a thrombotic angiopathy involving the epicardial veins of the atrial appendage was the only evidence that the patient may have had an underlying prothrombotic state, and its prognostic significance could only be fully appreciated after the development of a pulmonary embolism.

## Case Description

An 87-year-old Caucasian woman with mitral valve regurgitation and atrial fibrillation was presenting for surgical management. Medical history was notable for hypertension. Medication list was notable for lisinopril, clopidogrel, and aspirin. Laboratory testing was notable for a complete blood count showing a normocytic anemia, and coagulation studies were with reference range (prothrombin time of 11.9 s, INR of 1.1, and activated partial thromboplastin time of 31 s). A preoperative echocardiogram revealed a severely dilated left atrium, mild to moderately dilated right atrium, and severe mitral valve regurgitation. Coronary angiography showed no significant coronary artery stenosis. The patient would undergo a Cox-maze procedure, ligation and excision of the left atrial appendage, and mitral valve annuloplasty with a ring placed: the surgery was completed without complication and the patient would subsequently exhibit normal sinus rhythm.

**Figure 1:**
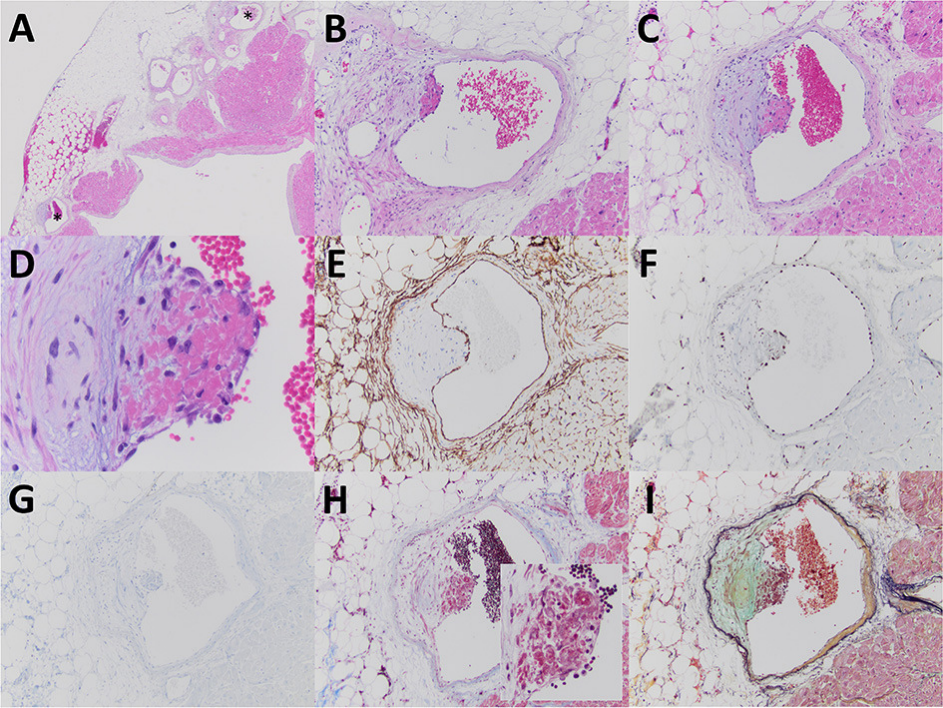
The excised left atrial appendage revealed few epicardial veins involved by a non-occlusive acute thrombotic angiopathy (**A–C**, with stars indicating veins at lower power magnification). The acute thrombotic angiopathy was characterized by eccentric intimal edema, endotheliosis (note swollen nature of endothelial cells adjacent to fibrin), and fibrin deposition **(D)**. The vessels involved were determined to be veins based upon morphologic features including an attenuated smooth muscle layer and lined by endothelial cells that by immunoperoxidase staining expressed CD34 **(E)** and ERG **(F)** and were negative for D2-40 **(G)**. Fibrin material associated with the endothelial injury was characteristically fuchsinophilic by Masson trichrome stain (**H** inset) and bright red by Russell-Movat pentachrome stain **(I)**. The usually encountered findings of endocardial fibroelastosis **(A)** and cardiomyocyte hypertrophy **(B,C)** were also evident. Images **(A–D)** are from paraffin sections stained with hematoxylin and eosin. Original magnification for **(A)** at ×20; for **(B,C)**, and **(E–I)** at ×100; for **(D)** at ×400; and for (**H** inset) at ×200.

Pathologic gross examination of the excised atrial appendage noted smooth and unremarkable pericardial and endocardial surfaces, without the presence of thrombus formation along the endocardial surface. Sections of the appendage were submitted for processing for light microscopy. Microscopic examination revealed the common findings of cardiomyocyte hypertrophy and endocardial fibroelastosis and also showed few epicardial veins with evidence of an acute thrombotic angiopathy characterized by intimal edema, endotheliosis, and overlying fibrin (Figure 1). The acute thrombotic angiopathy was non-occlusive, and there was no evidence of associated ischemic injury of adjacent tissues including myocardium. The epicardial veins that exhibited evidence of an acute thrombotic angiopathy were found in the mid-portion of the atrial appendage, with no evidence of such vascular/endothelial injury in epicardial veins near the line of excision or the distal tip.

The post-operative course was unremarkable, and the patient would be discharged 4 days after surgery on aspirin and clopidogrel.

**Figure 2:**
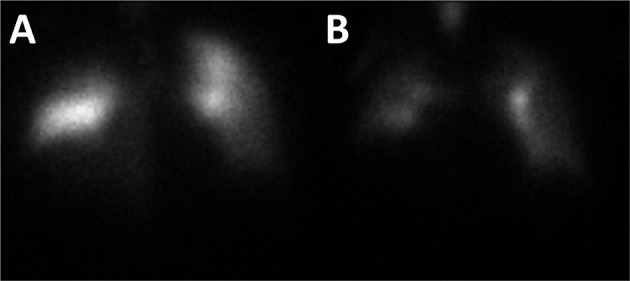
A ventilation and perfusion lung scan was performed. Analysis of the perfusion images revealed multiple mismatched segmental/subsegmental defects (particularly in the right lung) and matched non-segmental defects along the periphery of both lungs **(A)**. Analysis of the ventilation images revealed moderate heterogeneity of tracer deposition with retention of radiotracer in the central bronchi and several areas of diminished ventilation along the periphery of both lungs **(B)**. Both images are of the anterior view.

Three weeks after discharge while at home, the patient developed shortness of breath with mild chest pain and was transported to another institution. Based upon the patient's examination findings of dyspnea, decreased oxygen saturation, lack of history of significant coronary artery disease, and point-of-care testing for troponin I being below the reference range, there was a strong clinical concern for underlying pulmonary embolism. Ultrasound examination of the lower extremities did not reveal evidence of thrombosed veins. Laboratory testing from blood drawn at presentation would notably revealed D-dimer of 1,45,910 ng/ml and fibrinogen of 126 mg/dl. A ventilation and perfusion lung scan was performed and revealed findings consistent with acute pulmonary embolism (Figure 2). While being transported from the imaging study, the patient became suddenly hypotensive and pulseless. Unfortunately, the patient's status was recalcitrant to resuscitative measures and was declared dead. An autopsy was not requested by the patient's family.

## Discussion

Thrombotic angiopathy is a pathologic description to describe endothelial injury which in its mildest form is characterized by endotheliosis (endothelial swelling) but can with sufficient and sustained injury lead to exposure of underlying tissue factor and the deposition of associated fibrin material. Thrombotic angiopathy has been associated with thrombotic thrombocytopenic purpura, hemolytic uremic syndrome, hypercoagulable states including antiphospholipid syndrome, other autoimmune diseases, monoclonal proteins, exposure to toxins (including chemotherapeutic agents), radiation, in the setting of malignant neoplasms, allogeneic hematopoietic stem cell transplantation, etc., (4). Direct physical trauma (i.e., catheter induced) has been infrequently associated with endothelial injury and thrombotic angiopathy developing along the endothelial-lined surfaces in the heart (5). As in the presented patient, a definitive underlying etiology may not be elucidated; however, thorough clinical examination and laboratory work-up are necessitated.

An etiology for the finding of an acute thrombotic angiopathy involving the epicardial veins of the left atrial appendage in this case is not clear. While the turbulent flow within the fibrillating atrial appendage is a primary risk factor for the development of a thrombus within the chamber, data suggests that the prothrombotic milieu within the appendage also potentiates thrombus formation (6, 7). Bartus et al. illustrated that from direct sampling of blood from within the atrial appendage compared with sampling from other heart chambers revealed a local prothrombotic state with evidence of prolonged clot lysis time and reduced clot porosity (6). Others have also described increased levels of procoagulant factors within the atrial appendage compared with the right atrium and femoral vein, further supporting local activation of the coagulation system (7). These described factors producing a prothrombotic state within the atrial appendage chamber albeit are likely not pertinent for the observed epicardial venous changes present in our patient. Although the patient did not have a history of clinically evident manifestations of thrombotic angiopathy or laboratory evidence (based on the limited coagulation studies performed preoperatively) of a procoagulant/prothrombotic state, the fact that the patient showed evidence of differing tissue sites (atrial appendage and pulmonary arteries) involved by sequelae of a prothrombotic state and a thrombotic angiopathy suggests a systemic etiology for the epicardial vein findings.

Common histopathologic changes found in excised atrial appendages in the setting of atrial fibrillation include cardiomyocyte hypertrophy, interstitial fibrosis, and endocardial fibroelastosis, with thrombus formation within the chamber identified more commonly within the left vs. the right appendage (1). In the largest review of surgically excised atrial appendages (including review of bilaterally excised appendages), Castonguay et al. did not document the finding of epicardial venous acute thrombotic angiopathy/thrombosis in the reviewed specimens. The finding of epicardial venous thrombosis has only been described in the English language literature by Hansen who noted the finding in 16 of 50 autopsy cases of patients with acute myocardial infarction (8). In the presented case, the epicardial venous findings were not in association with significant coronary artery disease or acute myocardial infarction. Within our own institution, the finding of epicardial venous acute thrombotic angiopathy/thrombosis was limited to the presented case in a review of the past 101 examined surgically excised atrial appendages (incidence rate of <1%, with a 95% confidence interval of 0.17–5.4%).

Excised atrial appendages are relatively common specimens examined by pathologists particularly at medical centers with cardiovascular surgery services (1), and generally they reveal regularly encountered histopathologic changes (*vide supra*). The finding in the present case of atrial appendage epicardial vein involvement with an acute thrombotic angiopathy has not been described previously, and the description of such a process involving any epicardial vein is exceedingly rare in the literature (8). Although the presented patient developed fatal sequelae of a prothrombotic state, the prognostic significance of the rarely described finding of epicardial vein involvement by an acute thrombotic angiopathy is not clear. Further studies closely examining the prevalence of acute thrombotic angiopathy within epicardial veins of excised atrial appendages are required to better understand the mechanism of formation and what prognostic significance is imparted.

## Data Availability Statement

The original contributions presented in the study are included in the article/supplementary material, further inquiries can be directed to the corresponding author/s.

## Ethics Statement

The patients provided written informed consent for publication of any potentially identifiable images or data in this article.

## Author Contributions

All authors listed have made a substantial, direct and intellectual contribution to the work, and approved it for publication.

## Conflict of Interest

The authors declare that the research was conducted in the absence of any commercial or financial relationships that could be construed as a potential conflict of interest.

## References

[1] Castonguay MC Wang Y Gerhart JL Miller DV Stulak JM Edwards WD. Surgical pathology of atrial appendages removed during the cox-maze procedure: a review of 86 cases (2004 to 2005) with implications for prognosis. Am J Surg Pathol. (2013) 37:890–7. 10.1097/PAS.0b013e31827e180b 23629441

[2] Nishikii-Tachibana M Murakoshi N Seo Y Xu D Yamamoto M Ishizu T. Prevalence and clinical determinants of left atrial appendage thrombus in patients with atrial fibrillation before pulmonary vein isolation. Am J Cardiol. (2015) 116:1368–73. 10.1016/j.amjcard.2015.07.055 26358509

[3] American College of Cardiology Foundation, American Heart Association, European Society of Cardiology, Heart Rhythm Society Wann LS Curtis AB. Management of patients with atrial fibrillation (compilation of 2006 ACCF/AHA/ESC and 2011 ACCF/AHA/HRS recommendations): a report of the American College of Cardiology/American Heart Association Task Force on practice guidelines. Circulation. (2013) 127:1916–26. 10.1161/CIR.0b013e318290826d 23545139

[4] Izzedine H Perazella MA. Thrombotic microangiopathy, cancer, and cancer drugs. Am J Kidney Dis. (2015) 66:857–68. 10.1053/j.ajkd.2015.02.340 25943718

[5] Lopez JA Ross RS Fishbein MC Siegel RJ. Nonbacterial thrombotic endocarditis: a review. Am Heart J. (1987) 113:773–84. 3548296 10.1016/0002-8703(87)90719-8

[6] Bartus K Litwinowicz R Natorska J Zabczyk M Undas A Kapelak B. Coagulation factors and fibrinolytic activity in the left atrial appendage and other heart chambers in patients with atrial fibrillation: is there a local intracardiac prothrombotic state? (HEART-CLOT study). Int J Cardiol. (2020) 301:103–7. 10.1016/j.ijcard.2019.09.053 31787387

[7] Fuster V Ryden LE Cannom DS Crijns HJ Curtis AB Ellenbogen KA. ACC/AHA/ESC 2006 Guidelines for the Management of Patients with Atrial Fibrillation: a report of the American College of Cardiology/American Heart Association Task Force on Practice Guidelines and the European Society of Cardiology Committee for Practice Guidelines (Writing Committee to Revise the 2001 Guidelines for the Management of Patients With Atrial Fibrillation): developed in collaboration with the European Heart Rhythm Association and the Heart Rhythm Society. Circulation. (2006) 114:e257–354. 10.1161/CIRCULATIONAHA.106.177292 16908781

[8] Hansen BF. Thrombosis of epicardial coronary veins in acute myocardial infarction. Am Heart J. (1979) 97:696–700. 433746 10.1016/0002-8703(79)90003-6

